# Supplier Risk Assessment—A Quantitative Tool for the Identification of Reliable Suppliers to Enhance Food Safety Across the Supply Chain

**DOI:** 10.3390/foods14081437

**Published:** 2025-04-21

**Authors:** Sina Röhrs, Sascha Rohn, Yvonne Pfeifer, Anna Romanova

**Affiliations:** 1 SGS Germany GmbH, Health and Nutrition, Heidenkampsweg 99, 20097 Hamburg, Germany; sina.roehrs@sgs.com (S.R.); yvonne.pfeifer@sgs.com (Y.P.); 2 Department of Food Chemistry and Analysis, Institute of Food Technology and Food Chemistry, Technische Universität Berlin, Gustav Meyer Allee 25, 13355 Berlin, Germany; 3 SGS Estonia Ltd., Vana-Narva mnt. 27A, 74114 Maardu, Estonia; anna.romanova@sgs.com

**Keywords:** food safety, artificial intelligence, supplier risk assessment

## Abstract

Food safety is a global issue that can be enhanced by collaboration with reliable suppliers. Given the complexities of international supply chains, identifying reliable suppliers is often challenging and resource-intensive. Integrating artificial intelligence (AI) offers a valuable opportunity to improve efficiency in this process. The aim of the present study was to develop a quantitative supplier assessment scheme for implementation in an AI-supported database. The framework developed incorporates different indicators, including the hazard risk, incident category level, vulnerability of a commodity, audit performance, logistic performance index, gross domestic product (GDP) growth, and GDP per capita. Each indicator is evaluated according to its own distinct assessment. Ultimately, the sub-assessments are integrated into the calculation of a supplier’s overall risk score. Hereby, it is possible to set individual weightings for each indicator. Manual testing using an exemplary selected supplier yielded promising results, indicating that the next steps involve implementation into an AI-supported database. It can be concluded that such an assessment framework can be an effective method for the identification of reliable suppliers. A future challenge will be to establish incentives to make audit data freely available, as these are often restricted and cannot be considered in the supplier risk assessment.

## 1. Introduction

According to estimates from the *World Health Organization*, approximately 600 million individuals globally suffer each year from diseases resulting from the consumption of contaminated food [1]. To decrease this number, enhancing food safety and compliance with applicable regulations and laws is a critical global concern. Recently, a food issue of a big fast-food chain caused attention after numerous consumers fell ill following the consumption of *E. coli*-contaminated onions [2]. Those onions were sourced from a facility where inspections by the *U.S. Food and Drug Administration* (FDA) identified concerns regarding sanitation, including unclean equipment, insufficient handwashing protocols, and inadequate cleaning procedures. This incident underscored the essential role of reliable suppliers to maintain safety and quality standards throughout the whole food supply chain.

However, identifying reliable suppliers can be highly challenging due to complex global supply chains, varying supplier practices, and inconsistent regulatory compliance across the global economic areas (e.g., the European Union vs. the United States Mexico Canada Agreement—USMCA). Limited data and rapid changes in procurement can also obscure potential risks. Nevertheless, it is crucial for companies to evaluate the potential risks associated with a supplier prior to procuring goods from them. In this context, a supplier’s risk refers to the probability of distributing food that is contaminated and unsafe for (human) consumption. Therefore, it is important for companies to conduct a comprehensive regular supplier risk assessment to monitor, identify, evaluate, and mitigate potential risks in their individual supply chain, ensuring continuity, compliance, and reliability in the procurement process.

However, this process can be highly intricate. According to Art. 14 (1) Reg (EC) No. 178/2002, food shall not be placed on the market if it is unsafe and Art. 17 mentions that food business operators shall ensure that food satisfies the requirements of food law at all stages of production, processing, and distribution [3]. As companies typically engage with multiple suppliers within different markets for varying raw materials, ingredients, and products, monitoring compliance can be highly complex. The specific regulatory requirements to be fulfilled often vary depending on the individual goods and markets involved. Moreover, the specific analyses required to ensure food safety may vary depending on the stage of the food supply chain. For instance, in the case of raw materials, the parameters to be analyzed are different to those that are applicable to processed food products. This is attributable to the fact that the subsequent processing steps can result in the formation of new, potentially hazardous process contaminants.

Another area of concern arises from practices that compromise food safety in favor of maximizing supplier profits. Food fraud is an emerging area in the field of food safety that is gaining more and more recognition and awareness. However, food fraud can also be quite diverse. It refers to the intentional misrepresentation, adulteration, or tampering of food products for financial profit [4]. Product tampering involves the modification of product characteristics such that they no longer align with the implicit or explicit claims associated with the product. Different processes fall under the category of product tampering. On the one hand, there is the application of undeclared or unapproved processes to increase the perceived quality or to hide deficiencies of a product. An example for the latter would be the injection of excessive water into raw meat to augment its weight and volume [5]. On the other hand, the intentional removal of a substance expected to be present in a product, while continuing to sell the remaining product at its original full price despite its diminished value, also constitutes an act of product tampering. An example of this act of tampering involves the separation of cream, a valuable component of milk, intended for sale as whole milk. The producer’s failure to accurately label the modified product as skimmed or reduced-fat milk, instead falsely marketing it as whole milk at the original price, constitutes a clear example of food tampering [6]. The opportunities for food fraud to enhance supplier profits are diverse, and their implications for food safety can range from minor to severe, depending on the specific nature of the violation. This underscores the critical importance of closely monitoring and rigorously overseeing such violations by suppliers.

To evaluate the reliability of a supplier, companies can employ a variety of data sources and assessment methodologies. Such a monitoring process may begin with an evaluation of the supplier’s reputation and experience of other industry partners and further extends to a review of potential incident reports related to the supplier of interest and its products. This can be achieved, for instance, through an online survey, including platforms such as the *Rapid Alert System for Food and Feed* (RASFF) in the EU, as well as by consulting other stakeholders like trade associations who have prior experience with specific suppliers. To facilitate a more detailed supplier evaluation, audits at the supplier’s production facilities are often already part of a cooperation and procurement contracts. This approach enables on-site assessment of adherence to hygiene standards and the prevailing working conditions. In this context, factors such as food manufacturing processes and storage conditions can be checked directly. Furthermore, pilot projects and test deliveries can be negotiated with a supplier to evaluate factors such as product quality and delivery reliability prior to establishing a formal contractual business relationship. However, this is only reflecting an actual status without getting an impression of past issues or incidents closely related to that supplier. Moreover, such measures demand substantial costs and human resources, as the manual collection of information and the execution of audits are time-intensive and laborious processes. This indicates that long-term contracts and traditional as well as well-established supply chains are more advantageous. However, due to the growing complexity and volatility of the markets, established supply chains are decreasing, and it becomes more and more challenging to keep all aspects in perspective.

The current era of technological advancement and digitalization, with possibilities of handling large data sets or even employing artificial intelligence (AI), provides novel and more efficient opportunities in the field of food safety and for the implementation of a regularly in-house supplier risk assessment. As reviewed by Yu et al., the integration of AI offers many opportunities to improve food safety [7]. For instance, AI is already used in food monitoring as well as in the analysis of images and the evaluation of data received from sensors utilized for quality control in the food supply chain [8,9,10,11]. Moreover, AI can be highly effective in optimizing the utilization of the continually growing volume of available data. In this context, an initial approach to the efficient and time-saving risk assessment of food incidence reports has been developed, facilitating the effective utilization of large datasets [12]. Röhrs et al. evaluated the risk associated with incidence reports based on a pre-defined assessment framework. In that study, an evaluation scheme was designed in such a way that it can be implemented in existing AI-supported databases. These can serve as a platform offering comprehensive data resources for food safety.

An AI-supported database, such as Digicomply (SGS DIGICOMPLY, Chiasso, Switzerland), offers extensive capabilities for the efficient acquisition of information, as outlined previously. A dataset encompasses various sources, including legal texts, scientific articles, and incidence reports [13]. For instance, an AI-supported database allows for the rapid and reliable determination of maximum residue limits for specific risks in distinct markets. The potential applications of the resources of AI-supported databases are vast and multifaceted. They can be used for the advantageous utilization of large volumes of food safety data originating from sources like RASFF, enabling the early identification of potential emerging food safety risks [12].

Although the development of a risk assessment scheme is initially time-intensive, the subsequent time savings for all kinds of users/stakeholders are undeniable. To gain an effective approach, it is essential to supply the AI-supported systems with all the necessary information, and detailed workflows and algorithms need to be established to enable it to perform the risk assessment automatically and independently. The use of AI can provide advantageous support, but its effectiveness is limited by the quality and accuracy of the information. Besides Digicomply, there are similar platforms available with their own individual foci. For instance, the Medical Information System (MediSys, European Commission, Brussels, Belgium) serves as a tool for media monitoring, enabling the early detection of potential health risks. It systematically analyzes media reports on topics such as infectious diseases, food and feed contaminations, and chemical or biological threats, autonomously generating alerts for stakeholders, including regulatory authorities [14]. In Germany, the system BeoWarn (The Federal Office of Consumer Protection and Food Safety (BVL), Berlin, Germany) is used by official authorities of the different federal states. It serves as an early detection and monitoring system for potential risks in the food sector. It integrates data from multiple sources, including RASFF, the *Emerging Risks Exchange Network* (EREN) of the *European Food Safety Authority* (EFSA), as well as risk assessments conducted by the *Federal Institute for Risk Assessment* (BfR), Berlin, Germany. However, users from the private sectors are not able to use BeoWarn for e.g., a reliability evaluation of suppliers.

Beyond the systems/platforms mentioned, a commercially platform known as FOODAKAI (Agroknow IKE, Athens, Greece) is available to mitigate food safety risks. It systematically collects and processes data on potential hazards associated with raw materials and food products, enabling the early identification and assessment of risks [14].

Some of the existing platforms provide a solid foundation for developing and implementing additional applications to be used in the context of food safety evaluations. The risk assessment of suppliers seeks to systematically document and evaluate food safety issues, in a thorough and comprehensive manner. However, the risk assessment framework for incidence notifications, initially developed to be implemented in the platform Digicomply, offers a valuable starting point for further research and development [12]. It can be also effectively applied in a similar form for the risk assessment of suppliers. The platform integrates over 3000 validated sources, providing food safety information collected from more than 160 countries [13]. More than 5 million entries, including legal requirements, incidence notifications, the titles and abstracts of scientific publications, and social media news, are available [13]. The data undergo systematic verification and are updated on a daily basis. The platform utilizes ML/AI tools with a group of six classifiers (type, market, topic, product category, incident category, and hazard category) that are able to understand the general scope of different types of documents like articles and notifications [13]. Moreover, six named-entity recognition models are utilized to extract specific information from the different data sources [13]. Each of these models undergoes continuous updates and enhancements to ensure optimal accuracy and performance. By the platform’s crawlers, identified content is processed with ML/AI models and made available immediately. Additional details regarding the characteristics of the AI-supported database utilized are comprehensively explained by Röhrs et al. [13]. In their paper, the comprehensive data resources were described while the subsequent paper addresses the development of an AI-supported assessment scheme for the risk evaluation of incidence reports [12]. Different databases, like the RASFF or a database from the FDA, are freely available to monitor information on food safety incidents. The data utilized by the AI-supported database are primarily publicly accessible and can be viewed by any individual. However, without the AI-supported database, it would be necessary to visit each source and website individually to search for the desired information, like incidence notifications. With the assistance of the AI-supported database, the information is consolidated, allowing the user to save time by utilizing a single database, rather than visiting and reviewing each page individually. Additional time can be saved when the incidence reports are pre-assessed and categorized by the AI, making it immediately apparent which reports pose a risk and require further examination [12]. AI-supported data evaluation can significantly reduce time demands, making it beneficial to expand the evaluation framework for incidence reports to include additional areas of application. To implement further applications, it will be necessary to consider further data resources compared with the studies carried out by Röhrs et al., where mostly information incidence notifications were considered [12,13].

It is hypothesized that an AI-supported supplier risk assessment can also be established, which allows for the efficient and rapid processing of large volumes of data, facilitating the quick identification of reliable or non-recommendable suppliers. Therefore, the objective of this study was to develop a supplier risk assessment procedure, similar to the one that was developed for evaluating novel risks [12].

## 2. Materials and Methods

The supplier risk assessment should be integrated into the same AI-supported database intended for the risk assessment of the incidence reports [13]. To enable the AI to independently perform the supplier risk assessment developed herein, relevant information and detailed workflows must first be defined to ensure that the AI will be able to make reliable decisions, even in varying situations.

**Figure 1:**
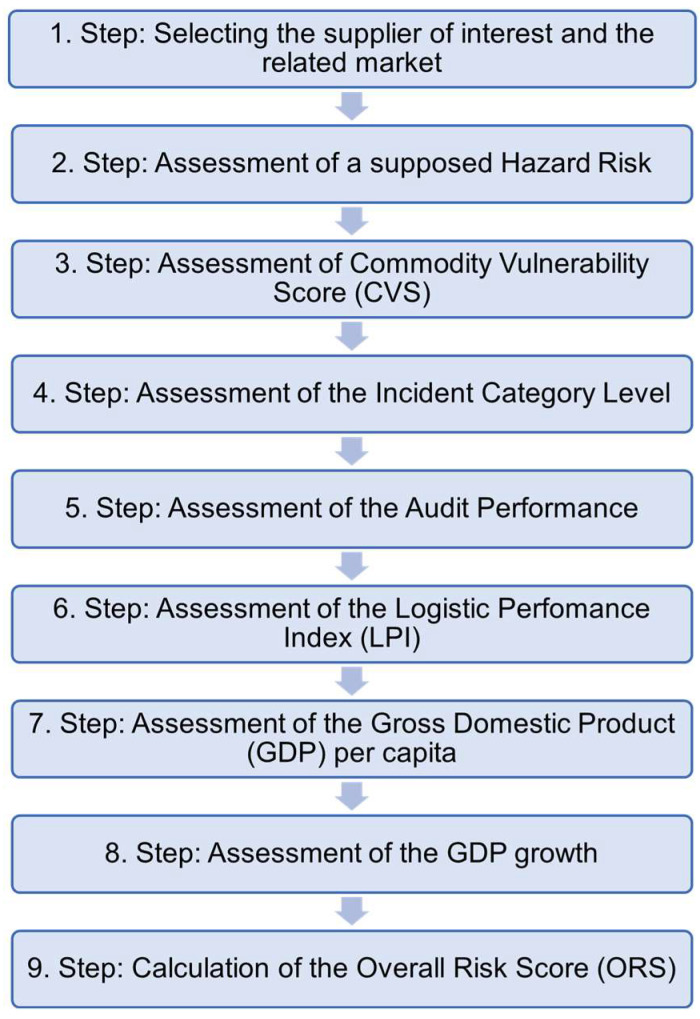
Workflow describing the single steps of the supplier risk assessment.

The supplier risk assessment is carried out according to the following scheme (Figure 1).

To perform the supplier risk assessment, the supplier under evaluation and the corresponding market are initially selected. As previously described, many companies engage with multiple suppliers across various markets. However, to ensure that the final application in an AI-supported database can precisely identify the relevant supplier data, the supplier must be specified by name at the outset of the supplier risk assessment process. When necessary, the commodity of interest can also be defined. Subsequently, the risk associated with a specific supplier will be assessed. The risk will be evaluated through the assessment of seven different indicators. The indicators suggested here can offer insights across various domains regarding the reliability of a supplier. The indicators to be evaluated are presented in Figure 1 (Step 2–Step 8). Moreover, further details on the potential relevance of distinct indicators are provided in the corresponding sections of the subsequent methodology.

### 2.1. Hazard Risk

Food incidence reports are diverse and address a wide variety of events. The potential risk to the food consumer may vary, depending on the specific topic of the incident report. Therefore, not all food incident reports present an equally high risk for the consumer. Thus, it is crucial to differentiate incident reports based on their potential risk, allowing more hazardous incidents to be identified preferably, while less dangerous ones often may not necessitate further attention. To consider the differences in risk, the first indicator (hazard risk, cf. Step 2, Figure 1) aims to qualify the risk associated with the goods and products of a specific supplier. Ultimately, this approach can provide a comprehensive overview of whether a supplier has already had incident reports with potentially significant risk levels in the past or had no incidents at all.

The potential risk associated with the incident notifications concerning the supplier under evaluation will be assessed using a procedure that has already been described previously [12] (Table 1).

Once the risk assessment points have been assigned in accordance with the evaluation framework, the total risk can be calculated by weighting the overarching risk categories (1st-level weights) and the according associated subcategories. The categorization of the hazard risk categories (Table 2, left column) is based on the points aligned with the classification [12].

After the calculation of the potential risk arising from an incidence notification, the point value will be normalized to be included in the ultimate assessment of the overall risk score (ORS). As the seven indicators (Figure 1, Step 2–Step 8) under evaluation are usually expressed in different units (e.g., points, currency, percentage), the calculated values will be normalized to facilitate comparability across the data. To combine the data on different scales is often challenging to compare them directly. Normalization transforms the values into a standardized range between 0 and 1, enabling consistent comparisons. For the normalization, the value calculated for the hazard risk will be divided by 50, where 50 is the maximum achievable point value for this indicator (Table 2).

### 2.2. Commodity Vulnerability Score (CVS)

Certain products, such as raw chicken meat, are more prone to serious, health-threatening contaminations than other matrices. Therefore, the vulnerability of a commodity needs to be considered when assessing the overall risk. The CVS will be assessed considering the number of incidents within the past 12 months, reported by official governmental bodies such as the EU’s RASFF. A 12-month period was selected to ensure the inclusion of all seasonal variations, thereby mitigating the risk of overlooking seasonal fluctuations. For instance, when only the last 6 months of the year are considered, coinciding with the winter and spring seasons, bias in the results for certain product groups can be observed. In Germany, the majority of fruit and vegetable harvests occur during the summer and autumn seasons. This increases the likelihood of incidence reports for these product groups being recorded during these two seasons. When only the incidence reports from spring and winter were considered, it could create the false impression that there are fewer reports for fruits and vegetables than would be evident when data from the entire year were included.

For evaluating the vulnerability of a product group in comparison with other products, the number of incidents for a certain group of commodities will be divided by the total number of incidents within the past 12 months.

Commodity vulnerability = (Incidents for specific commodity/Total incidents) × 100

### 2.3. Incidents Category Level

Non-compliance with typical aspects of typical food safety quality control procedures can be identified at various stages throughout the supply chain. The risk of ingestion of contaminated goods by a consumer varies, depending on the stage at which the non-compliance is identified. When the non-compliance is detected prior to the goods entering circulation, the probability of consumption—and consequently the associated risk—is considerably lower compared with a scenario where the goods are already in circulation, and the non-conformity is only identified after a recall. When the non-compliance is detected while the goods are still e.g., on the ship or in storage, the products can be intercepted before reaching supermarkets and consumers. However, once contaminated goods have reached supermarkets, safeguarding consumers becomes more complex. In this case, some consumers may have already purchased and consumed the contaminated goods, meaning that the first individuals could have already been affected. Therefore, the incident category level (Figure 1, Step 4) is considered as an indicator in the supplier risk assessment. Based on the data available in the incident report, the timing of non-conformity detection will be assessed.

Points ranging from 1 to 4 will be assigned based on the timing of the non-conformity detection. A maximum of 4 points may be assigned based on the assessed severity to the end consumer. Table 3 outlines various scenarios of non-compliance detection and the corresponding severity levels, quantified in points. The points that could be assigned correspond to the following severities: 1 point: low severity; 2 points: low to medium severity; 3 points: medium to high severity; 4 points: high severity.

Once the incident reports have been assessed concerning the timing of the non-conformity detection, the assigned points are summed and divided by the total number of reports evaluated. This yields the following formula:

Incident category = Total risk points/Total incident categories

Exemplarily, the calculation of the incident category level for a supplier (“Supplier A”) may proceed as follows:

Example calculation—Supplier A’s incidents:

For example, according to published incident reports, the non-conformities of a supplier may have been identified during three recalls, two outbreaks, and one control. In this case, the incident category level would be calculated as follows:

3 recalls = 3 incidents × 3 points = 9 points

2 outbreaks = 2 incidents × 4 points = 8 points

1 control = 1 incident × 1 point = 1 point

Total risk points: 9 (recalls) + 8 (outbreaks) + 1 (control) = 18 total points

Total incident categories = 3 (recalls) + 2 (outbreaks) + 1 (control) = 6 incidents

Incident category = Total risk points/Total incident categories = 18/6 = 3

The exemplarily calculated risk score is 3. However, to be incorporated into the calculation of the ORS, this value has to be normalized in advance. Table 4 illustrates the classification and normalization of the calculated risk score. A calculated risk score of 3 would be assigned as moderate risk, meaning a normalized score of 0.7 would be considered in the calculation of the ORS.

### 2.4. Audit Performance

Where available, incorporating audit performance data is also recommended. An audit is a systematic evaluation of records, processes, or systems to ensure accuracy, compliance, and efficiency [21]. For instance, with regard to food safety, audits evaluate the adherence to legal and regulatory requirements and the compliance with quality standards (e.g., ISO certificates).

Audit data can yield critical information regarding product quality, safety, and regulatory compliance. For example, audit data provide detailed information on adherence to hygiene protocols, quality benchmarks, and international standards (such as ISO), facilitating the identification of process weaknesses and areas for optimization. For instance, when an audit reveals that a supplier is not adhering to hygiene regulations, it can serve as an early warning signal of potential food safety issues.

However, there might be confidentiality issues. Nonetheless, audit data would be a useful source, as they can be very specific with regard to products, but also internal procedures. The independent upload of the audit would technically cause no problem. Users of the AI-supported database can upload data independently and integrate them into the supplier risk assessment. Further, specific agreements or prerequisites can be demanded by a certain branch or distribution chain.

Based on the supplier’s performance in the audit, three levels of non-conformity can be distinguished (Table 5).

In the absence of audit performance data for a supplier, the assessment can still be conducted. However, when data are available, they should be incorporated, as it enhances the accuracy and reliability of the assessment. The more data that are considered, the more accurate the supplier risk assessment will be.

### 2.5. Economic Insights from the Supplier’s Country

Economic insights can reveal data on a country’s economic stability, which can help to determine whether the economic environment of a supplier poses a risk. Some countries often face more pronounced challenges related to food safety, including a higher frequency of food-related incidents and less comprehensive regulatory frameworks. These factors can contribute to increased risks in food safety and quality control.

Various indicators can be utilized for the economic evaluation of a supplier based on its country of origin. Here, the logistic performance index (LPI), the gross domestic product (GDP) per capita, and the GDP growth were considered.

#### 2.5.1. Logistic Performance Index (LPI)

The LPI index is published by the World Bank and evaluates a country’s logistics infrastructure as well as its logistics services, considering different criteria. On the one hand, these include the customs and border management by assessing their effectiveness: the LPI highlights how smoothly goods, including food products, move across borders. On the other hand, the quality of the infrastructure is considered: the LPI evaluates the quality of the logistics infrastructure, such as ports, roads, and warehousing facilities. Moreover, the LPI considers the competence of logistics services, including transportation providers and logistics companies. High-quality and transparent logistics contribute to the better management of food safety and the reduced risk of incidents. Additionally, the LPI measures the efficiency of a country’s transportation networks and the ability to track and trace shipments throughout the supply chain. Taking all these factors into consideration, the World Bank calculates the LPI index for 139 countries worldwide and makes the results publicly accessible [22].

The index ranges from 1 to 5, with a higher score representing a better performance. To be incorporated in the final suppliers’ risk assessment, the LPI index will also be normalized, as follows.

LPI normalization = (5 − LPI score)/4

#### 2.5.2. Gross Domestic Product (GDP) per Capita

GDP per capita is an economic indicator that measures a country’s output relative to its population size. It is calculated by dividing the country’s total GDP by its population number, providing an average economic output per person. This is a fundamental measure of economic well-being and the standard of living. Developing countries with a lower GDP per capita often have weaker food safety regulations and infrastructure, leading to a higher probability of food incidents. The *World Bank* provides a comprehensive overview of GDP per capita from countries all over the world [23].

Based on the GDP per capita of the supplier’s country, an assessment is conducted and classified into one of three categories (Table 6). Here, a normalization is carried out as well. Depending on the assigned category of the GDP per capita (high, moderate, or low), the corresponding normalized value (0.3, 0.7, or 1.0) will be included in the final calculation of the supplier’s risk.

#### 2.5.3. GDP Growth

GDP growth refers to the increase in a country’s GDP over time, which measures the total value of the goods and services produced within the country during a specific period, typically on a quarterly, semi-annual, or annual basis. GDP growth is commonly expressed as the percentage change in GDP from one period to the next, reflecting the extent of economic expansion or contraction. For instance, an expanding economy is marked by a positive GDP growth. Different factors, like net exports, are considered for the calculation of the GDP growth. When the exports of a country are higher than the imports, the net exports contribute positively to GDP growth, while a trade deficit may impede it. Moreover, governmental investments in infrastructure, education, and healthcare or industrial investments in machinery and technology can stimulate economic growth. For instance, the infrastructure is vital for transporting food from producers to consumers, while minimizing losses during harvesting, storage, and transportation. Improved access to education leads to the development of a more skilled workforce for the food industry, ultimately enhancing food safety through expertise in e.g., food hygiene. The *World Bank* provides a comprehensive overview of the GDP growth from countries all over the world [24].

Depending on the GDP growth, an assignment to one of four categories and, thereby, to the corresponding normalized value is carried out (Table 7).

### 2.6. Calculation of the Overall Risk Score

After the assessment of the seven individual indicators, the ORS of a supplier will be calculated. Table 8 summarizes the described procedure for the seven indicators and the assigned weightings.

The ORS will be calculated as a weighted average of the risk and its associated weight. The calculation will be carried out using the following formula:

Overall risk score = (I_1_ × W_1_ + I_2_ × W_2_ + I_3_ × W_3_ + I_4_ × W_4_ + I_5_ × W_5_ + I_6_ × W_6_ + I_7_ × W_7_) × 100

where:

I_i_ is the risk of the indicator (normalized from 0.0 to 1.0) and W_i_ is the weight associated to the indicator.

**Figure 2:**
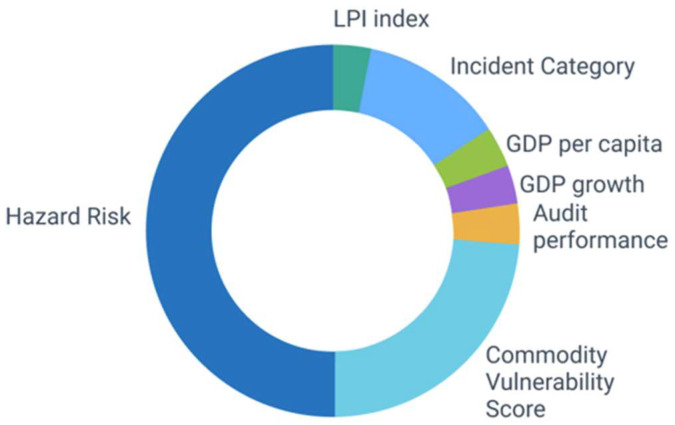
Graphical illustration of the individual distribution of weightings for the different indicators of the supplier risk assessment.

The seven identified indicators are incorporated into the ORS calculation with variable weightings, while the sum of the individual weights must add up to 100% (Figure 2).

**Figure 3:**
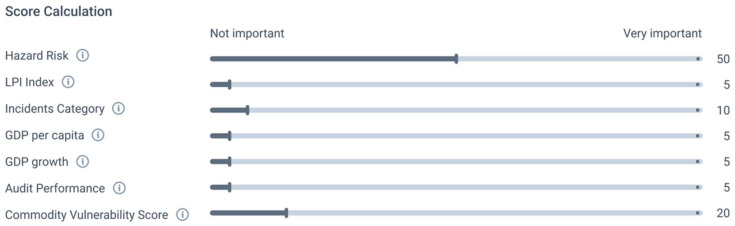
Individual distribution of weightings for the different indicators of the supplier risk assessment.

The weightings provided in Table 8 are intended as a recommendation. However, the AI-supported platform, which finally handles the supplier risk assessment, also allows for the individualized adjustment of the weightings (Figure 3).

After the calculation of the ORS, the result is categorized into one of three supplier risk categories (Table 9).

The 33.63% lower limit of the risk classification is based on the lowest possible ORS for the sum of the seven risk indicators and the weightings of each indicator. Table 10 outlines the minimum possible values for the seven indicators utilized in the supplier risk assessment.

Calculation of the minimum overall risk score

Overall risk score = (I_1_ × W_1_ + I_2_ × W_2_ + I_3_ × W_3_ + I_4_ × W_4_ + I_5_ × W_5_ + I_6_ × W_6_ + I_7_ × W_7_) × 100 = (0.5 × 0.54 + 0.2 × 0.00007692 + 0.1 × 0.3 + 0.05 × 0.3 + 0.05 × 0 + 0.05 × 0.3 + 0.05 × 0.125) × 100 = 33.63%

When no data are available for one or more indicators, the assigned weight will have to be distributed proportionally among the remaining indicators to ensure that the total weight remains equal to 100%.

For example, when the LPI and the GDP per capita are missing (each 5%), this weight would be distributed proportionally across the other categories. Mathematically, the distribution of the 10% would be as follows:

Exemplary calculation:
  Scaling factor: 100%/90% = 1.111

Therefore:

Hazard risk (50%) → 55.55%; Incident category (10%) → 11.11%; GDP growth (5%) → 5.56%; Audit performance (5%) → 5.56%; Commodity vulnerability (20%) → 22.22%.

## 3. Results

To assess the feasibility and applicability of the developed supplier risk assessment, the framework will initially be evaluated manually through the example of a supplier with a food fraud issue:

In September 2024, *Food Standards Scotland* (FSS) issued a warning to consumers about counterfeit vodka products, particularly those fraudulently labeled as a traditional Scottish trademark product, following recent seizures in Coatbridge, Scotland and Glasgow, Scotland. The counterfeit vodka, sold in 35 cl-bottles, posed serious health risks due to the presence of toxic isopropyl alcohol, which can lead to severe symptoms or even death.

To perform a risk assessment of this supplier, the developed assessment framework would be applied as follows:

### 3.1. Hazard Risk

The points assigned in Table 11 will now be incorporated into the calculation of the hazard risk.

Risk categorization = R_1_ × P_1_ + R_2_ × P_2_ + R_3_ × P_3_ + R_4_ × P_4_ = 1 × 10 + 1 × 3 + 2 × 6 + 6 × 4 = 49

With a total of 49 points, this kind of hazard is classified into the high-risk category (Table 2). After normalization (c.f. Section 2.1), a result of 0.98 can be noted, which will subsequently be incorporated into the calculation of the supplier’s risk.

### 3.2. Commodity Vulnerability Score for Counterfeit Vodka Products

In the past 12 months, there were five incidents involving vodka products. In total, 12,431 incidents were reported in the past year. Using the formula in Section 2.2, a CVS of 0.04% results, indicating a low vulnerability, because there were only a few incidents.

Commodity vulnerability = (5/12,431) × 100 = 0.04%

### 3.3. Incident Category Level

The incident report concerning the counterfeit vodka product addresses the presence of toxic isopropyl alcohol, necessitating its classification within the fraud incident category (Table 3).

1 food fraud incident = 1 incident × 3 points = 3 points

Total risk points: 3 (food fraud)

Incident category = Total risk points/Total incident categories = 3/1 = 3

The calculated incident category level is 3 and according to Table 4, the corresponding normalized value is 0.7, which will finally be incorporated into the calculation of the supplier’s risk.

### 3.4. Audit Performance

In this example, no audit data were available for this supplier. Consequently, this indicator will not be further incorporated into the calculation of the overall supplier’s risk.

### 3.5. LPI

The company of interest is located in the United Kingdom. The LPI score is 3.7. To be incorporated in the final supplier’s risk assessment, the LPI index will also be normalized.

LPI normalization = (5 − LPI score)/4 = (5 − 3.7)/4 = 0.325

The normalized value of 0.325 will be incorporated into the calculation of the overall supplier’s risk.

### 3.6. GDP per Capita

In 2023, the GDP per capita of the United Kingdom was $49,463.9. Therefore, it will be categorized into the high-GDP-per-capita category and the value for the incorporation into the final supplier’s risk is 0.3 (Table 6).

### 3.7. GDP Growth

In 2023, the GDP growth for the United Kingdom was 0.3% and will be categorized as low growth (Table 7). The corresponding normalized value to be incorporated into the final supplier’s risk is 0.7.

### 3.8. Calculation of the ORS

For the final calculation of the overall supplier’s risk, the individual values of the seven indicators will be incorporated into the calculation of the ORS.

In the absence of audit data, the weightings for the remaining six indicators must be adjusted. The 5% weighting originally allocated to the potential audit data will be redistributed proportionally among the six remaining indicators.

Scaling factor: 100%/95% = 1.0526

Therefore, the new weightings of the remaining six indicators are as follows:

Hazard risk (50%) → 52.63%; Commodity vulnerability (20%) → 21.052%; Incident category (10%) → 10.526%; LPI (5%) → 5.263%; GDP growth (5%) → 5.263%; GDP per capita (5%) → 5.263%.

Overall Risk Score = (I_1_ × W_1_ + I_2_ × W_2_ + I_3_ × W_3_ + I_4_ × W_4_ + I_5_ × W_5_ + I_6_ × W_6_ + I_7_ × W_7_) × 100 = (0.98 × 0.5263 + 0.04 × 0.21052 + 0.7 × 0.10526 + 0.325 × 0.05263 + 0.3 × 0.05263 + 0.7 × 0.05263) × 100 = 66.79%

I_1_ × W_1_ = Weighted hazard risk; I_2_ × W_2_ = Weighted commodity vulnerability score; I_3_ × W_3_ = Weighted incident category level; I_4_ × W_4_ = Weighted audit performance: not included as no data are available; I_5_ × W_5_ = Weighted LPI; I_6_ × W_6_ = Weighted GDP per capita; I_7_ × W_7_ = Weighted GDP growth.

The calculated ORS for this exemplary supplier is 66.8%, classifying it as moderate-risk supplier based on the categorization criteria outlined in Table 9.

In addition, the risk assessment scheme was applied to 10 further suppliers. The results and details of the individual suppliers are listed below in Table 12 and Table 13.

For legal reasons, specific supplier names cannot be disclosed. However, since the aim of this paper was to develop a method for the supplier risk assessment that can be integrated into an AI-supported database following initial testing, rather than to publicly challenge the reputation of individual suppliers, the inclusion of specific names is not required.

## 4. Discussion

The supplier risk assessment is an advantageous tool for evaluating suppliers across multiple criteria, facilitating systematic risk categorization. The manual test run was successful, allowing for the next step of completing the implementation within an overall AI-supported database (here: Digicomply) and enabling the supplier risk assessment to operate autonomously and independently. The results presented in Table 13 demonstrate the feasibility of conducting a differentiated risk assessment of suppliers using the proposed evaluation framework. The comparative analysis clearly indicates that the evaluated suppliers with the lower food safety risks were classified as low risk based on the calculations. In contrast, all the other evaluated suppliers were assigned to the moderate-risk category. However, it should be noted that a disruption in the cold chain, as observed with Supplier H, can rapidly escalate into a more severe food safety risk. While classification as a low-risk supplier may be justified in the context of a disrupted cold chain, the potential consequences of such an event should not be overlooked. Inadequate cooling of food can lead to spoilage caused by microbiological factors, including the proliferation of pathogenic organisms, such as *Salmonella* spp. and *E. coli*. In the event of such contamination, the consumption of the affected products poses a significant health risk to the consumer. In such cases, the limitations of the assessment scheme become apparent, highlighting the need for a final human evaluation of both the risk assessment and its underlying data in order to account for potential broader consequences that may not be anticipated by the AI system. Moreover, an evaluation of the results in Table 13 reveals that certain suppliers are on the threshold of being classified as high risk, based on their assessment scores. Given the food risk levels assigned to the suppliers, it raises the question of whether categorizing them within the high-risk category would have been more appropriate. For instance, particularly for Supplier B, which received a rating of 69.6%, the result is marginally close to the threshold for classification as a high-risk supplier. Had Supplier B been based in Somalia rather than the United States, the variables in the calculation would have been altered, resulting in a total risk assessment of 75.7%, which would have classified it as a high-risk supplier. This demonstrates that even minor changes in the variables can significantly alter the resulting risk categorization. This situation should be particularly considered when the calculated overall risk is near the threshold of an adjacent risk category, as demonstrated in the case of Supplier B. Considering the health risks associated with *E. coli* ingestion, classifying this supplier as high risk would have been more appropriate in this case. This serves as another example where the AI and the assessment scheme reach their limitations, and a final judgment should be rendered by a human evaluator. This example also illustrates the impact that the weighting of individual indicators can have on the overall risk assessment. Even with a relatively low weighting of 5% for the LPI, GDP per capita, and GDP growth, these indicators can ultimately prove decisive in determining the final result. This factor should be carefully considered when assigning weightings to the individual indicators.

Having statistical data on the suppliers enables companies to make informed decisions about which supplier in which country to choose. Factors such as the suppliers’ performance in audits or the number of incidents associated with the supplier need to be considered. The exemplary application of the supplier risk assessment classified a moderate-risk supplier in a case of “only” food fraud. However, reaching a risk level that is also presenting a serious health risk not often is quite easy.

Another example of varying risks is bananas. At the time of harvest, the products are generally in good condition, but still green, which means they are non-edible. However, no immediate health risk can be assumed initially. After harvesting, the bananas continue to ripen by applying ethylene during transportation and storage [25]. The ripening process can affect their edibility when, for example, the ripening process is too fast, leading to unwanted browning. So, the quality of the final (yellow) product is quite sensitive, requiring a high awareness from the supplier. Another potential risk associated with bananas could be the inadvertent transport of venomous spiders [26]. All in all, bananas are a good example of a raw product that is safe at the time of harvest but may develop potential risks within the course of transport and ripening, ranging from low risks (partly brown bananas) to very severe health risks (spider venom).

For the exemplary assessed supplier in this manuscript, it would be prudent to explore alternative suppliers for potential collaboration, as the assessment revealed a moderate risk. The utilization of the supplier risk assessment, along with the associated quantitative comparability of the suppliers, enhances regulatory compliance pressure, thereby contributing to improved food safety. The pressure on suppliers to comply with legal requirements increases, as a poor performance compared with competitors in suppliers’ risk assessments may lead to customer loss and a consequent decline in sales. The supplier risk assessment facilitates the rapid, quantitative, and, when using AI, an automated comparison of suppliers. This approach makes it straightforward for companies to identify underperforming suppliers and replace them with those demonstrating a superior performance. This, in turn, can encourage suppliers to maximize their performance in the suppliers’ risk assessment. When suppliers show a certain transparency and align more closely with regulations, such as hygiene requirements, for continuously maintaining and improving their risk assessment scores, it enhances overall food safety.

To achieve a high accuracy in a supplier risk assessment, it is recommended to evaluate multiple indicators. However, due to the current limitations in data availability, a broader selection of indicators is often not yet fully achievable. For instance, the evaluation of the audit performance presents particular challenges [27]. In this context, the non-restricted availability of this partially sensitive data remains highly limited. From the perspective of the audited supplier, the withholding of audit data, although understandable, is not surprising. On the other hand, being as transparent as possible would lead to more trust and reliability. It is therefore not unexpected that a supplier with a poor audit performance would be reluctant to disclose this sensitive data publicly. However, the selective disclosure of audit data—where information on poor performance is withheld while data on strong performance are shared—would distort the results and hinder the ability to achieve the most accurate supplier risk assessment possible. There are undoubtedly different approaches to drive the disclosure of audit data. This could be achieved either voluntarily by suppliers or through the implementation of legal regulations or significant pressure by the customer. A highly consistent, though challenging, approach would be the legal requirement to make all audit data freely accessible. This would present major challenges, particularly in terms of data protection. However, there could also be a smoother approach that relies more on voluntary participation within a limited set of framework conditions. For instance, some audit data from specific suppliers can already be accessed and reviewed [28]. In this respect, the German QS database provides information on QS-certified suppliers within the food supply chain, including audit records and monitoring results [29]. However, this platform is limited to information on QS-certified suppliers and companies, and not all the data are free and accessible, most are only available for authorized individuals like auditors or participating suppliers. Overall, the informational value of the audit data for the supplier risk assessment should not be underestimated. Therefore, enhancing the accessibility of audit data is a future key objective.

However, ensuring the free accessibility of data is not the only challenge in conducting supplier risk assessments. It is also necessary to critically evaluate the informational value of individual indicators, such as GDP growth. As mentioned in Section 2.5.3, GDP growth can serve as a measurable indicator for the availability of financial resources, which can facilitate investments in education and infrastructure to enhance food safety [30,31]. In principle, this represents a reliable indicator for evaluating a country’s capacity to allocate resources toward initiatives, such as skilled labor training, which might further contribute to enhancing food safety.

GDP growth provides insight solely into the current economic situation of the respective country [31]. As this value is incorporated into the supplier risk assessment to account for the availability of financial resources for areas such as education and infrastructure, the broader context of the country’s overall situation in these domains should also be considered. The benefits of investments persist over several years, as infrastructures such as roads and harbors remain functional beyond a single year, and skilled workers retain their expertise over time. GDP growth, on the other hand, provides insight only into one-year periods. A negative GDP growth rate may indicate that there are currently insufficient financial resources for investment in education and infrastructure; however, it does not offer any information regarding the current state of a country’s infrastructure or education network. When the country in question has demonstrated economic growth in previous years, enabling investment, the situation is considerably less critical than in the case of a country that has experienced economic deficits over an extended period. For instance, Germany’s GDP growth for 2023 was −0.3%, while Ethiopia, for example, experienced a growth rate of 6.5% [24]. In the supplier risk assessment, this would lead to a bad rating for a German supplier and a good rating for an Ethiopian one. However, this approach does not account for the fact that Germany has already made substantial investments in education and infrastructure due to its historically strong economic performance. Although Ethiopia is currently experiencing a strong GDP growth, it lacks the economic history of Germany, which allowed it to develop a high-quality education and infrastructure system. Therefore, when considering GDP growth, it is important to recognize that it reflects only a one-year value; it does not provide direct insights into a country’s educational and infrastructural conditions. Investments in these sectors are long-term endeavors that cannot be realized in a short period. Based on this consideration, a negative GDP growth in Germany may not be as critical as it initially appears, while a positive GDP growth in Ethiopia may be less promising than it seems. Overall, it is crucial to interpret GDP growth within the context of the specific country in question.

In addition to the seven factors currently considered in the supplier risk assessment, additional indicators may be integrated in the future. For instance, an additional potential indicator could be meteorological data. Meteorological conditions can elevate the risk of food contamination. In the current era of climate change, with varying weather conditions and even events like floods and droughts, there are improved conditions for food contamination with mycotoxins [32]. For example, mycotoxin synthesis can be initiated by a range of environmental and biological factors, sometimes serving as a defense mechanism in response to environmental stressors. These stressors may encompass suboptimal storage conditions, with variations in humidity and temperature, but also droughts or insect infestations [33]. The growth of mycotoxin-producing molds is often facilitated by a moist and warm environment with an adequate supply of nutrients [34]. Therefore, incorporating the environmental conditions in which a supplier’s products were grown, harvested, or stored may provide valuable insights when assessing the supplier’s risk. It could be argued that the supplier has no direct influence over the meteorological conditions and that this indicator would pertain to the risk associated with the product rather than the supplier itself. However, this situation is comparable with the commodity vulnerability score: while the product may be at risk, the supplier remains ultimately responsible for ensuring the safety of their goods. Although incorporating weather data into the supplier risk assessment is logically justified, its practical implementation presents a challenge. For example, to enable accurate implementation, precise weather data would be needed. Although data loggers are available, it is very laborious and needs a certain accurateness in handling. Additionally, weather stations can facilitate the collection of meteorological data. However, it is a complex issue overall, as the availability of such resources, in the form of weather stations, is not uniform across all regions of the world [35]. Additionally, processing these large volumes of data presents a challenge, but the use of AI could also be an effective solution for this issue.

## 5. Conclusions

The AI-supported supplier risk assessment is an advantageous tool that enables companies to make informed decisions about which supplier in which country to choose. The manual test run was successful, and the next step is to complete the implementation within an AI-supported database (here: Digicomply). However, situations may arise where the result is marginal, thereby exposing the limitations of both the AI and the assessment framework, thus requiring a final judgment by a human evaluator.

Notwithstanding, in the current era of global commodity flows, the quantitative assessment scheme enables the comparison of suppliers across various factors, which, in turn, should increase the pressure on suppliers to adhere to regulations and legislation, ultimately enhancing food safety. For the most accurate supplier risk assessments, it is essential to consider a comprehensive range of data. In this regard, the currently limited availability of audit data presents a major challenge. Particularly, audit data can offer valuable insights into the degree to which suppliers adhere to regulations and laws. Users of holistic AI-supported databases, which incorporate the supplier risk assessment, can upload available audit data; however, the availability of such data is highly limited because of confidentiality issues. Therefore, a key objective for the future is to improve the unrestricted availability of these data through voluntary or regulatory approaches.

A future challenge will be to enhance the supplier risk assessment by incorporating additional indicators, such as weather data, to improve accuracy through the inclusion of further information. Another indicator within the food supply chain that becomes increasingly important is the consideration of environmental and sustainability factors, like the reduction of carbon emissions [36]. In this context, it would be feasible to expand the supplier risk assessment by incorporating an additional indicator that evaluates the extent of supplier compliance with environmental and sustainability criteria.

A direct comparison with other tools and methods is challenging, as the AI-supported risk assessment of suppliers remains a relatively nascent field of scientific research, and the use of AI-supported databases is often not free of charge. However, to our knowledge, no existing tool possesses the same capabilities, which underlines the importance of the developed tool.

## Figures and Tables

**Table 1:** Framework for the risk evaluation of an incident report [12].

Criteria	1st-Level Weights	2nd-Level Weights
*Relevance of matrix*	1	
The incident notification addresses the product of interest		10
*Signal Category*	1	
*Safety*		4
*Compliance*		3
*Public Perception*		3
*Reason of notification*	2	
Exceeded limit value/positive results		6
New recommendation		4
*Health Risk*	6	
*Limited Health Risk*		1
*Moderate Health Risk*		2
*Serious Health Risk*		3
*Very Serious Health Risk*		4

**Table 2:** Normalization scale for the calculated potential hazard risk arising from an incidence notification.

Hazard Risk Categories	Normalization (0–1 Scale)
Negligible Risk: ≤31 points	Negligible Risk: points/50
Low Risk: 32–37 points	Low Risk: points/50
Moderate Risk: 38–43 points	Moderate Risk: points/50
High Risk: ≥43 points	High Risk: points/50

**Table 3:** Scenarios and corresponding risk points for the identification of food non-compliances.

Incident Category	Points	Explanation and Examples
Control	1	Low severity. The incident is managed before it becomes critical. For instance, the notification is initiated through a sample taken at a border post for analysis [15]
Recall	3	Medium to high severity as there is an immediate threat to consumer safety. For example, the notification is initiated when a company informs the competent authority of the results from its internal monitoring process [16]
Outbreak	4	High severity. The incident poses a direct public health risk. Reports of food poisoning resulting in the notification of a potential risk associated with a marketed food product linked to the illness of consumers [17].
Fraud	3	Medium to high severity, depending on the severity of the fraud. Examples are a report of a deliberate and fraudulent alteration, like the substitution of olive oil with sunflower oil or the unauthorized use of novel foods [18].
Consumer complaint	1	Low to medium severity, depending on the specific incident. For instance, the notification is initiated by a consumer reporting a complaint with the competent authority (e.g., insects in beans) [19].
Other incidents	2	Depending on the specific incident, the severity is low to medium. Everything that does not fall into the previous categories—because, for example, the data are not available or an appropriate assignment is not possible because of the category descriptions. e.g., the Thai department of livestock development order on the temporary suspension of the importation or transit of live domestic pigs and wild boars and their carcasses from Italy to prevent the spread of African swine fever virus [20]

**Table 4:** Classification and normalization of the calculated risk score of the incident category level.

Description	Risk Score	Normalization (0–1 Score)
1.00–1.75	Low risk	0.3
1.75–3.25	Moderate risk	0.7
3.25–4.00	High risk	1.0

**Table 5:** Level of audit non-conformities and the corresponding metrics for the calculation of the supplier’s risk.

Scoring Criteria	Scope	Metrics
Minor	A minor non-conformity is issued when the identified deviation does not compromise the overall effectiveness of the management system in achieving its intended objectives.	0.3
Major	A major non-conformity is issued when a detected deficiency impacts the effectiveness of the management system in achieving its intended objectives and ensuring compliance with established standards.	0.7
Critical	A critical non-conformity is identified when an observed deficiency during the audit poses an immediate risk to food safety without adequate corrective action by the organization or when legal compliance and/or certification integrity are compromised.	1

**Table 6:** GDP per capita categorization and the corresponding normalized value for the calculation of the supplier’s risk.

GDP per Capita	Normalization (0–1 Scale)
High GDP per capita: ≥$12,696	High: 0.3
Moderate GDP per capita: $1046–$12,695	Moderate: 0.7
Low GDP per capita: ≤$1045	Low: 1.0

**Table 7:** GDP growth categories and corresponding normalized value for the calculation of the supplier’s risk.

GDP Growth	Normalization (0–1 Scale)
High GDP growth: ≥6% annually	High growth: 0.125
Moderate GDP growth: 2–5% annually	Moderate growth: 0.42
Low GDP growth: ≤2% annually	Low growth: 0.70
Negative GDP growth: <0% annually	Negative growth: 1.0

**Table 8:** Summary of the seven indicators considered for the supplier risk assessment.

Indicator	Weight (Approximate Estimate)	Counting Method	Normalization (0–1 Scale)
Hazard risk	50%	Negligible risk: ≤31 points Low risk: 32–37 points Moderate risk: 38–43 points High risk: ≥44 points	Negligible risk: (≤31)/50 Low risk: (32–37)/50 Moderate risk: (38–43)/50 High risk: (≥44)/50
LPI index	5%	The index ranges from 1 to 5, with a higher score representing a better performance	LPI normalization = (5 − LPI score)/4
Incident category	10%	Low risk: 1.00–1.65 Moderate risk: 1.66–2.32 High risk 2.33–3.00	Low risk: 0.3 Moderate risk: 0.7 High risk: 1.00
GDP per capita	5%	High GDP per capita: ≥ $12,696 Moderate GDP per capita: $1046–$12,695 Low GDP per capita: ≤$1045	High: 0.3 Moderate: 0.7 Low: 1.00
GDP growth	5%	High GDP growth: ≥6% annually Moderate GDP growth: 2–5% annually Low GDP growth: ≤2% annually Negative growth: <0 annually	High growth: 0.125 Moderate growth: 0.42 Low growth: 0.70 Negative growth: 1.0
Audit performance	5%	Minor: 0.3 Major: 0.7 Critical: 1	Minor: 0.3 Major: 0.7 Critical: 1
Commodity vulnerability score	20%	Incidents for the past 12 months with a certain group of commodities (e.g., fresh fruits, cheese, vegetables, flours and starches…) Incidents for a specific commodity divided on total incidents	The higher the number, the more vulnerable the commodity is

**Table 9:** Supplier risk categorization of the calculated overall risk score.

Supplier Risk Categorization	Supplier of Low Risk	Supplier of Moderate Risk	Risky Supplier
Overall Risk Score	33.63% to 50%	50% to 70%	70% to 100%
Explanation	Minimal risk, likely manageable	Medium concern; requires monitoring/preventive measures	Critical concern; immediate action or mitigation required

**Table 10:** Minimum possible value for each of the seven indicators in the supplier risk assessment included indicators as well as the corresponding recommended weightings.

Indicator	Weight	Lowest Risk Value	Comments
Hazard Risk	50%	0.54	The lowest score is 27, after harmonization it becomes 0.54
CVS	20%	7.69 × 10^−5^	Assuming that at least one incidence report exists for the specific commodity for the past 12 months and per year, there are ~13,000 incidents, 1/13,000 = 7.69 × 10^−5^ After the implementation of the supplier risk assessment in the AI-supported database, the count for incidents for the past 12 months will not be approximate anymore but based on the database and adjusted on a daily basis
Incident Category	10%	0.3	
Audit Performance	5%	0.3	
LPI Index	5%	0	The maximum achievable value for the LPI is 5, after the normalization ((5-5)/4) it would be 0
GDP per Capita	5%	0.3	
GDP Growth	5%	0.125	

**Table 11:** Framework for the risk evaluation applied to the above-mentioned incident notification of counterfeit vodka products. The points assigned as part of the evaluation framework are marked with a thick black frame.

Criteria	1st-Level Weights	2nd-Level Weights
*Relevance of matrix*	1	
The incident notification addresses the product of interest		10
*Signal Category*	1	
*Safety*		4
*Compliance*		3
*Public Perception*		3
*Reason of notification*	2	
Exceeded limit value/positive results		6
New recommendation		4
*Health Risk*	6	
*Limited Health Risk*		1
*Moderate Health Risk*		2
*Serious Health Risk*		3
*Very Serious Health Risk*		4

**Table 12:** Information on the suppliers assessed in Table 13, including details on the reported incidents and the respective countries of origin for each supplier.

Supplier	Description
A	Supplier of alcoholic beverages from United Kingdom, glass fragments found in the bottles
B	Supplier of onions from the United States, *E. coli* outbreak
C	Supplier of alcoholic beverages from Australia, undeclared allergen
D	Supplier of ice cream from Spain, ingredient list not in Italian language
E	Supplier of sauce from Poland, undeclared allergen
F	Supplier of baby food from Malaysia, detection of aflatoxin B1
G	Supplier of baby food from Canada, potential contamination with *Cronobacter* spp.
H	Supplier of ice cream from Switzerland, cold-chain break
I	Supplier of ice cream from China, possible presence of plastic and metal
J	Supplier of pizza from Australia, metal pieces in the products

**Table 13:** Results of the risk assessment of the ten suppliers mentioned in Table 12.

Supplier Risk Assessment	Supplier									
	A	B	C	D	E	F	G	H	I	J
Hazard Risk	1	1	1	0.62	1	0.82	1	0.62	1	1
Commodity Vulnerability Score	0.005	0.05	0.005	0.004	0.007	0.0007	0.0007	0.004	0.004	0.0006
Incident Category Level	0.7	1.0	0.7	0.7	0.7	0.7	0.7	0.7	0.7	0.7
Audit Performance	-	-	-	-	-	-	-	-	-	-
LPI	0.325	0.3	0.325	0.275	0.35	0.35	0.25	0.225	0.325	0.325
GDP growth	0.7	0.42	0.42	0.42	0.7	0.42	0.7	0.7	0.42	0.42
GDP per capita	0.3	0.3	0.3	0.3	0.3	0.7	0.3	0.3	0.7	0.3
Overall Risk Score [%]	67.1	69.6	65.6	45.3	67.3	58.3	66.6	46.6	67.7	65.5
Risk categorization	m. r.*	m. r.	m. r.	low risk	m. r.	m. r.	m. r.	low risk	m. r.	m. r.

* m. r. = moderate risk.

## Data Availability

The original contributions presented in the study are included in the article, further inquiries can be directed to the corresponding author.
